# Unusual multiple dentigerous cysts evaluated by cone beam computed tomography: a case report on a non-syndromic patient

**DOI:** 10.1016/j.bjorl.2020.05.016

**Published:** 2020-06-15

**Authors:** Mariana Lobo Bergamini, Guilherme Trafani Sanches, Paulo Sergio Souza Pina, Ricardo Pimenta D’Avila, Alan Motta do Canto, Celso Massahiro Ogawa, Paulo Henrique Braz-Silva, Andre Luiz Ferreira Costa

**Affiliations:** a Universidade de São Paulo (USP), Faculdade de Odontologia, Departamento de Estomatologia, São Paulo, SP, Brazil; b Faculdade de Medicina do ABC, Santo André, SP, Brazil; c Universidade Cruzeiro do Sul (UNICSUL), Programa de Pós-Graduação em Odontologia, São Paulo, SP, Brazil; d Universidade de São Paulo (USP), Instituto de Medicina Tropical de São Paulo, Laboratório de Virologia, São Paulo, SP, Brazil

## Introduction

A dentigerous cyst is defined as a cavity lined with epithelium originating from the expanded follicle located around the crown of an impacted tooth at the amelocemental junction. The cyst develops from the epithelial remnants of the enamel as a result of fluid accumulation within its strata.^1^

Dentigerous cysts are usually asymptomatic, but may cause an increase in volume and delayed tooth eruption.^1^, ^2^ They are routinely found on radiographic examinations for investigation of tooth eruption failure, tooth absence or malignancies.^1^ Radiographically, the lesion is usually expansive, unilocular, radiolucent and well-circumscribed, presenting a halo of reactive sclerotic bone associated with the crown of an impacted tooth.^1^, ^3^

On microscopic examination, these cysts have a cavity filled with fluid and a thin epithelial lining with 2–6 layers of cuboidal cells, generally being defined as non-keratinised. The interface between epithelium and cystic capsule is smooth. In some cases, it is possible to observe the presence of mononuclear inflammatory infiltrate in the cystic capsule.^3^

In the majority of patients, dentigerous cysts present as a single lesion. When they are bilateral or multiple, they usually are associated with developmental syndromes or systemic diseases such as mucopolyssacharidosis, basal cell nevus syndrome, cleidocranial dysplasia and Maroteaux-Lamy syndrome. The occurrence of multiple dentigerous cysts in the absence of systemic disease is rare.^3^, ^4^, ^5^

The imaging findings show a unilocular radiolucent lesion linked with an unerupted tooth with well-defined sclerotic margins. More sophisticated image diagnosis, such as computed tomography, may reveal the cystic nature of these lesions in greater detail and reliability, which facilitates the diagnosis.^6^

The aim of the present report is to describe an unusual case of multiple dentigerous cysts and to provide an overview of the imaging findings in order to assist dentists in performing accurate analysis of these lesions.

## Case report

A 39-year-old male patient presented to the dental office for conventional dental treatment, reporting neither underlying diseases nor oral complaints. Extra-oral examination showed no changes, whereas intra-oral examination showed no inflammatory signs.

**Figure 1:**
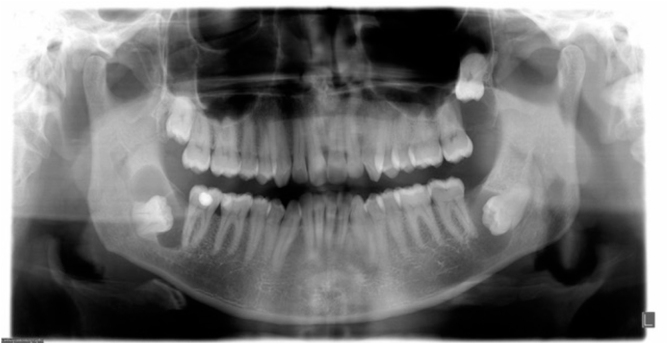
Panoramic radiograph showing cystic area around the unerupted teeth in three quadrants.

The panoramic radiograph showed the presence of two bilateral mandibular lesions, one measuring approximately 2 cm located on the right side and other measuring approximately 1.5 cm on the left side, and one maxillary lesion measuring 1.0 cm located on the left side. All of the three lesions were asymptomatic, well-delimited and involved the crowns of unerupted third molars (Fig. 1).

To determine the actual limits of the lesions and their relationships with the surrounding tissues, and consequently the best treatment approach, a Cone Beam Computed Tomography (CBCT) scan was performed.

**Figure 2:**
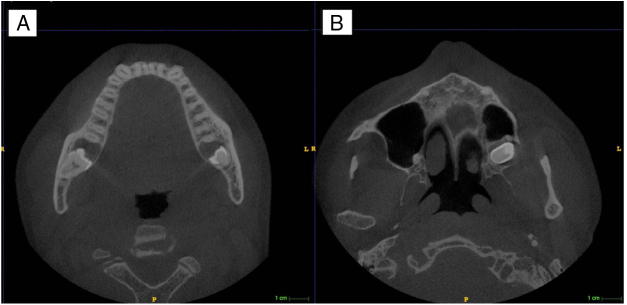
CBCT axial scans (A and B) showing bucco-lingual expansion and the cortical thinning in the mandible involving the third molars (A) and the upper left third molar (B).

**Figure 3:**
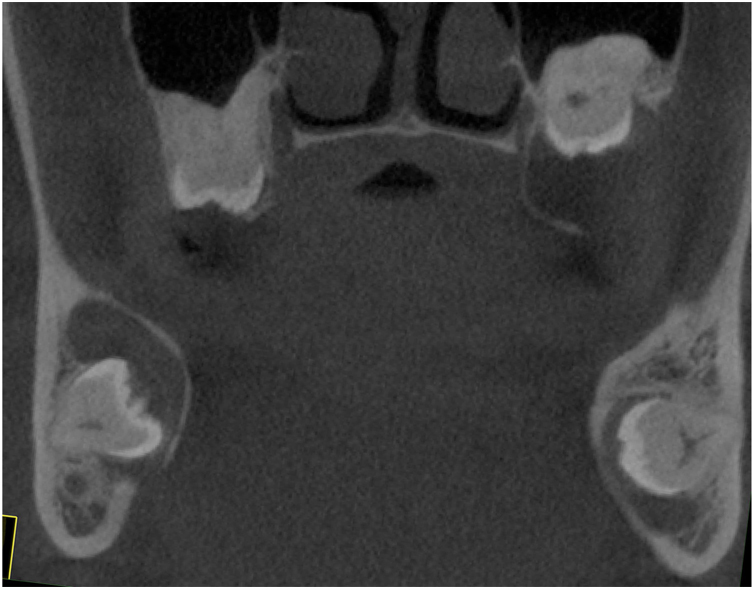
Coronal CBCT image showing expansive cystic lesion surrounding the crowns of impacted molars in maxilla and mandible.

CBCT revealed that teeth #28, #38 and #48 were unerupted, as well as the presence of cystic lesions on the right maxillary hemi-arch and on both mandibular right and left hemi-arches. In addition, CBCT (axial view) showed integrity and expansion of buccal cortical bones of maxilla and mandible (Fig. 2). Coronal CBCT image showing well-defined lesions with soft tissue attenuation involving impacted teeth (Fig. 3).

**Figure 4:**
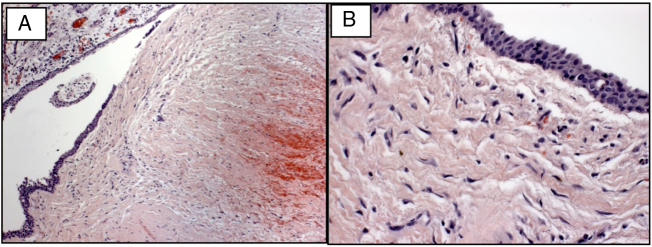
Hematoxylin-eosin stain showing histopathological features of dentigerous cyst. (A) Presence of cystic cavity with thin, stratified, squamous, epithelial lining (100× magnification); (B) interface between epithelial lining and smooth cystic capsule (400× magnification).

Clinical and radiographic findings were suggestive of odontogenic keratocyst and dentigerous cyst. Under local anesthesia, incisional biopsy of the lesions was performed and specimens obtained. The surgical specimens were stored in flasks containing 10% formaldehyde before being sent for histopathological examination in our pathology laboratory. Histopathological findings showed the presence of a cystic cavity with a thin, stratified, squamous, epithelial lining. The interface between epithelial lining and cystic capsule was smooth, presenting areas with significant extravasation of red blood cells. A diagnosis of dentigerous cyst was thus determined (Fig. 4).

The lesions were excised under general anaesthesia and the unerupted teeth removed.

## Discussion

A dentigerous cyst is the second most common type of odontogenic cyst, whose frequency is lower than that of root cyst.^6^ As for developmental odontogenic cysts, their frequency is the highest. They develop by means of fluid accumulation between enamel epithelium and the tooth crown.^1^

The treatment of dentigerous cyst is selected depending on the lesion’s size. The lesion can be enucleated if it is small, whereas marsupialisation can be necessary for complete removal of a larger cyst.^7^ Dentigerous cysts may involve impacted, unerupted permanent teeth, supernumerary teeth and rarely deciduous teeth. The mandibular third molar and maxillary canines are involved most frequently.^1^, ^2^, ^5^ Teeth adjacent to the lesion may be displaced or may suffer root resorption. However, these findings are not exclusive to this injury. Odontogenic keratocysts and odontogenic tumors may be radiographically similar.^2^ In the present case, however, no changes in surrounding teeth were observed. The lesion was well-delimited and involved the crowns of the impacted third molars.

In general, dentigerous cysts are asymptomatic, but may cause facial swelling and delayed tooth eruption. Also, a discomfort can be associated with the cyst once it is secondarily infected.^2^, ^8^ In the present case, the patient reported no pain symptoms and the lesions were only found radiographically. It is possible that these lesions would not have been revealed, had they not been noted on the panoramic radiography, until they became large enough to cause symptoms.

The multiple occurrence of dentigerous cysts in a non-syndromic patient is very rare.^9^ A comprehensive search for related articles on PubMed database revealed that, between the years 1943 and 2016, only 32 cases of bilateral dentigerous cysts in non-syndromic patients were reported.^5^

In the literature, age range varies widely, from 3 to 57 years old, occurring more in children under the age of 15 years.^4^ The patient was an adult, not having sought medical assistance due to the slow, asymptomatic cyst growth and lack of pain or discomfort. Batra et al. suggest that karyotyping should be performed to confirm the association with chromosome 1 anomaly or other chromosomes.^10^

The present case report demonstrates the necessity of radiographic examination to investigate unerupted teeth. All the teeth affected by the lesion were unerupted.

Some lesions may share the same radiographic features of dentigerous cyst, such as odontogenic keratocyst and unicystic ameloblastoma. However, dentigerous cysts typically have a smooth periphery whereas odontogenic keratocysts typically have a scalloped periphery.^4^, ^9^ Odontogenic keratocysts show a more discrete bone expansion and are less likely to cause tooth dental resorption.

In addition, findings may differ by imaging method, with certain modalities more suited for visualized the features of the cysts.^7^ For instance, extension of the lesion is not clearly seen on panoramic radiograph, but is well characterized on computed tomography, additionally providing information about origin, size, content, cortical plates and relationship of the lesion to adjacent anatomical structures.^9^

The complementary use of CBCT is important to delineate the extension of the lesion as well as its relationship with adjacent structures, provides brings more security to the surgical approach. CBCT has become widely used for diagnosis of the dento-maxillo-facial area. CBCT is a rapid, convenient and necessary measure that may be used to evaluate the presence and extent of this kind of lesion.^6^

## Conclusion

In conclusion, multiple dentigerous cysts very rarely affect normal individuals. Imaging findings are typically helpful for a proper diagnosis. This kind of lesion requires a comprehensive preoperative evaluation along with a logically chosen surgical approach, and CBCT as a newer imaging technique may be a the diagnostic tool of choice in those cases.

## Conflicts of interest

The authors declare no conflicts of interest.
